# The perfect storm: human influence on the loss potential of Eunice-like cyclones

**DOI:** 10.1088/1748-9326/ae886a

**Published:** 2026-07-20

**Authors:** Nicholas J Leach, Shirin Ermis, Aidan Brocklehurst, Dhirendra Kumar, Alexandros Georgios, Lukas Braun, Mark Dixon, Justin Murphy, Len Shaffrey

**Affiliations:** 1 Atmospheric, Oceanic, and Planetary Physics, University of Oxford, Oxford, United Kingdom; 2 National Centre for Atmospheric Science, Atmospheric, Oceanic and Planetary Physics, University of Oxford, Oxford, United Kingdom; 3 Climate X Ltd, London, United Kingdom; 4 Aon Impact Forecasting, London, United Kingdom; 5 National Centre for Atmospheric Science, Department of Meteorology, University of Reading, Reading, United Kingdom; 6 School of Climate Change and Sustainability, Azim Premji University, Bengaluru, India; 7 Cotality UK, London, United Kingdom; 8 National Oceanography Centre, Southampton, United Kingdom

**Keywords:** extreme weather attribution, impact attribution, insurance, extreme weather, climate change, European windstorms, weather forecasting

## Abstract

Storm Eunice was a severe windstorm that impacted Central Europe in February 2022. The meteorology and synoptic dynamics of Eunice have been studied in depth in several studies examining features of the storm such as its sting jet. The contribution of climate change to the storm dynamics and severity was examined in previous work, which found that in counterfactual weather forecasts—given an identical initial synoptic setup—climate change had measurably increased the severity of the storm. Here we move beyond meteorological attribution and quantify the role of climate change in the insured losses incurred during Eunice in, to the best of our knowledge, the first impact attribution of its kind for a European windstorm event. We combine the same counterfactual weather forecasts with three loss models, including two state-of-the-art commercial models, finding that the increases in meteorological severity do translate through to significant increases in estimated loss. We estimate a conditional increase in insured loss of nearly €2 bn between pre-industrial and present-day climates. Of particular note is the existence of several members within the forecast ensembles whose losses are far greater than what unfolded in reality. This includes one realisation, simulated in a warmer ‘future’ climate, in which the estimated loss could reach over 10x the realised loss during Eunice. The plausible existence of such a catastrophic loss is of considerable relevance to a wide variety of stakeholders across adaptation planning and the financial sector. We suggest that our results practically demonstrate not only the utility of counterfactual weather forecasts in quantifying impacts attributable to climate change, but also the value of academic—private partnerships in which the two sectors are able to bring different areas of expertise.

## 1. Introduction

Considerable progress has been made over the past two decades in understanding the impact of climate change on extreme weather (Seneviratne *et al* 2021). In particular, advances have been made in quantifying the human contributions to individual weather events through extreme event attribution (Allen 2003, Stott *et al* 2004, 2016, Mudhar *et al* 2024). There are a wide variety of motivating factors behind this work, including scientific understanding (Schumacher *et al* 2022), providing evidence in legal contexts (Allen 2003, Marjanac and Patton 2018, Burger *et al* 2020), informing loss and damage funding allocations (Wehner *et al* 2022, Mora *et al* 2025), informing decision makers over adaptation and mitigation policy (Zhang *et al* 2024), and the communication of climate change impacts to the public (Gärtner and Schoen 2021). The relevant information needed in the majority of these applications is not necessarily the attributable changes in extreme weather—but how those changes propagate through to socioeconomic impacts (Noy *et al* 2024).

Extreme event attribution has typically focussed on the weather itself as a necessary step to understanding the impacts, and statements regarding impacts have been qualitative. However, more recently, studies have explicitly attempted to calculate attributable changes in impacts such as incurred losses or excess mortality (Mitchell *et al* 2016, Frame *et al* 2020, Lott *et al* 2021). There are two main approaches that have been developed for this purpose: using meteorologically derived fractions of attributable risk (FAR) to partition the total cost of a particular event (Allen 2003, Frame *et al* 2020, Lott *et al* 2021, Newman and Noy 2023); or forcing impact models (which can be statistical or dynamical) using meteorological simulations (Wehner and Sampson 2021, Perkins-Kirkpatrick *et al* 2022). The FAR approach is the simplest, but its use in assessing the attributable costs of individual extreme weather events has been criticised due to the implicit binary impact definition and consideration of the event in question as one of a class (Perkins-Kirkpatrick *et al* 2022, Brown 2023). However, impact models bring novel challenges. Impacts often occur on very local scales and are non-linearly related to their physical drivers. As such, large-scale biases or local errors arising from coarse resolution in the climate models conventionally used for attribution can be amplified, resulting in large errors in any quantitative impact attribution statements. Downscaling and bias correction procedures (Thrasher *et al* 2024) may alleviate these issues, but statistical approaches introduce their own problems by breaking physical consistency or altering the underlying climate response if not used carefully (Maraun *et al* 2017). They are also unable to correct model biases in large-scale dynamical circulation (Shaw *et al* 2024).

Recent work has suggested that re-initialised weather forecast models could be used to advance impact attribution (Hope *et al* 2022, Leach *et al* 2024). Centring our approach around weather forecast simulations brings a number of relevant advantages: for a wide variety of weather events, they are typically far less biased than the climate models more conventionally used in attribution, reducing the need to apply unphysical statistical bias correction methodologies. They are also run at a higher resolution, improving the representation of localised weather extremes (Jung *et al* 2006, Roberts *et al* 2018, Schuster *et al* 2019). Finally, many impact modelling chains are already developed around weather forecast model output due to their importance in anticipatory action and emergency management (Alfieri *et al* 2013). In this work we demonstrate the application of weather forecasts in this context by translating previously produced counterfactual forecasts (Ermis *et al* 2024) into their associated impacts, allowing us to assess human influence on the direct economic impacts of an individual windstorm for the first time.

European windstorms are a considerable driver of natural hazard loss. They make up around 30% of the total and 50% of the insured loss from weather extremes across Europe (European Environment Agency 2025). The considerable damage they inflict results from their large size, with major windstorms typically impacting far larger areas than other hazards like floods. The damages themselves arise through a wide variety of channels, including: wind action through both gusts and sustained winds causing damage to roof tiles and the roof connection to building walls, potentially leading to roof failure; impact damage through airborne missiles such as light trees or fences, or through fallen larger trees; damage from water ingress; and wall failures resulting from both internal and external pressure. Wind gusts are highly related to such damages and are thus used as the key physical input to academic and industry loss models (Klawa and Ulbrich 2003, Kumar *et al* 2023, Cotality 2024, Aon Impact Forecasting 2025). While recent work suggests that precipitation intensity is also important for loss estimation (van Ederen *et al* 2025), the vast majority of insured loss models use wind gusts alone.

Storm Eunice was a severe windstorm that impacted central Europe in February 2022, causing over €2.5 bn in insured loss. It formed on a cold front west of the Azores before undergoing explosive cyclogenesis and tracking across Central Europe, producing measured wind gusts of up to 55 ms^−1^. It has been suggested that these exceptional wind speeds were caused by a range of physical mechanisms including a sting jet (Volonté *et al* 2024a, 2024b). The question of how intense extratropical cyclones are responding to climate change is still open. Due to the complex physical mechanisms underlying their formation and evolution (Schultz and Vaughan 2011, Laurila *et al* 2021), and deficiencies in the ability of most climate models to represent these mechanisms (Shaw *et al* 2016), there remains uncertainty over how these storms will evolve throughout the 21st century. However, Ermis *et al* were able to estimate the impact of climate change on Eunice conditional on the synoptic conditions before the storm using counterfactual ensemble weather forecasts. They re-initialised an operational weather forecast model from initial conditions with anthropogenic influence removed from the ocean state to quantify the impact of climate change on the storm.

Here we use three insured loss impact models to translate operational and counterfactual climate weather forecast ensembles, initialised in the lead up to storm Eunice, into predicted loss distributions. The loss and weather forecast models and counterfactual forecast experiments are described in section 2. We then use these loss distributions to quantify the impact of climate change on the damages incurred by a ‘Eunice-like’ storm, understand the key drivers of loss, and explore the plausible limits of incurred losses in section 3. We finally provide concluding remarks on the importance of impact attribution, industry-academic partnerships, and suggest directions for further work in section 4.

## 2. Methods and models

### 2.1. Forecast model

The counterfactual forecast model setup is identical to that used in Ermis *et al* (2024). They used the European centre for medium-range weather forecasts’ (ECMWF) integrated forecasting system (IFS) CY47r3, which was operational at the time of Eunice. This system was able to predict the evolution of the storm prior to its formation, a key test in establishing confidence in the ability of the model to represent the necessary physical processes (Leach *et al* 2021). This model configuration has 51 ensemble members, a horizontal resolution of approximately 18 km (TCo639), 137 vertical levels, a model timestep of 12 min and is coupled to a 0.25 degree ocean model.

The ‘current climate’ forecast is taken from the operational configuration used at ECMWF. The pre-industrial and future climate forecasts are based on perturbing the initial ocean state and boundary CO_2_ concentrations used in IFS. The ocean state perturbations are derived by linearly fingerprinting observational data (Haustein *et al* 2017) and are representative of the estimated anthropogenic warming from 1850 to 1900 to the forecast initialisation time; the CO_2_ concentration perturbations are based on the observed change in CO_2_ concentrations between 1850 and 1900 and the forecast initialisation time. Here, the future climate state is represented by an equal but opposite perturbation to the pre-industrial perturbation (Ermis *et al* 2024, Leach *et al* 2024). More detail is provided in section S1 in the supplementary materials.

Three forecast lead times are used: −2 d, −4 d and −8 d. These represent a range of conditioning, from heavily conditioned on the specific dynamics and track of the observed storm, to more loosely conditioned on the predictable larger scale dynamics. An alternative framing is that at short lead times, the natural weather variability represented within the forecast ensembles is negligible, but as the lead time increases, more variability will be represented. Thus the use of several of lead times allows us to understand how any conclusions, and their associated level of confidence, are dependent on the amount of weather variability captured within the model ensembles. However, it is important to bear in mind that, even at the longest lead, while this variability represents a wide range of possible weather outcomes, it will not include any significant variation in longer-term modes of climate variability, such as the North Atlantic Oscillation (NAO).

### 2.2. Loss models

In this study, we use three ‘formal’ impact loss models (Kumar *et al* 2023, Cotality 2024, Aon Impact Forecasting 2025), and one ‘proxy’ loss model. The formal loss models (henceforth the Aon IF, Cotality, and NCAS models) are described here, the proxy model is described below. Such loss models (often referred to as catastrophe models) aim to estimate the overall windstorm risk to relevant assets (in this case insured physical infrastructure). This is generally done through combining the three components of risk: hazard, exposure and vulnerability (Office of the United Nations Disaster Relief Co-ordinator 1980).

**Figure 1.**
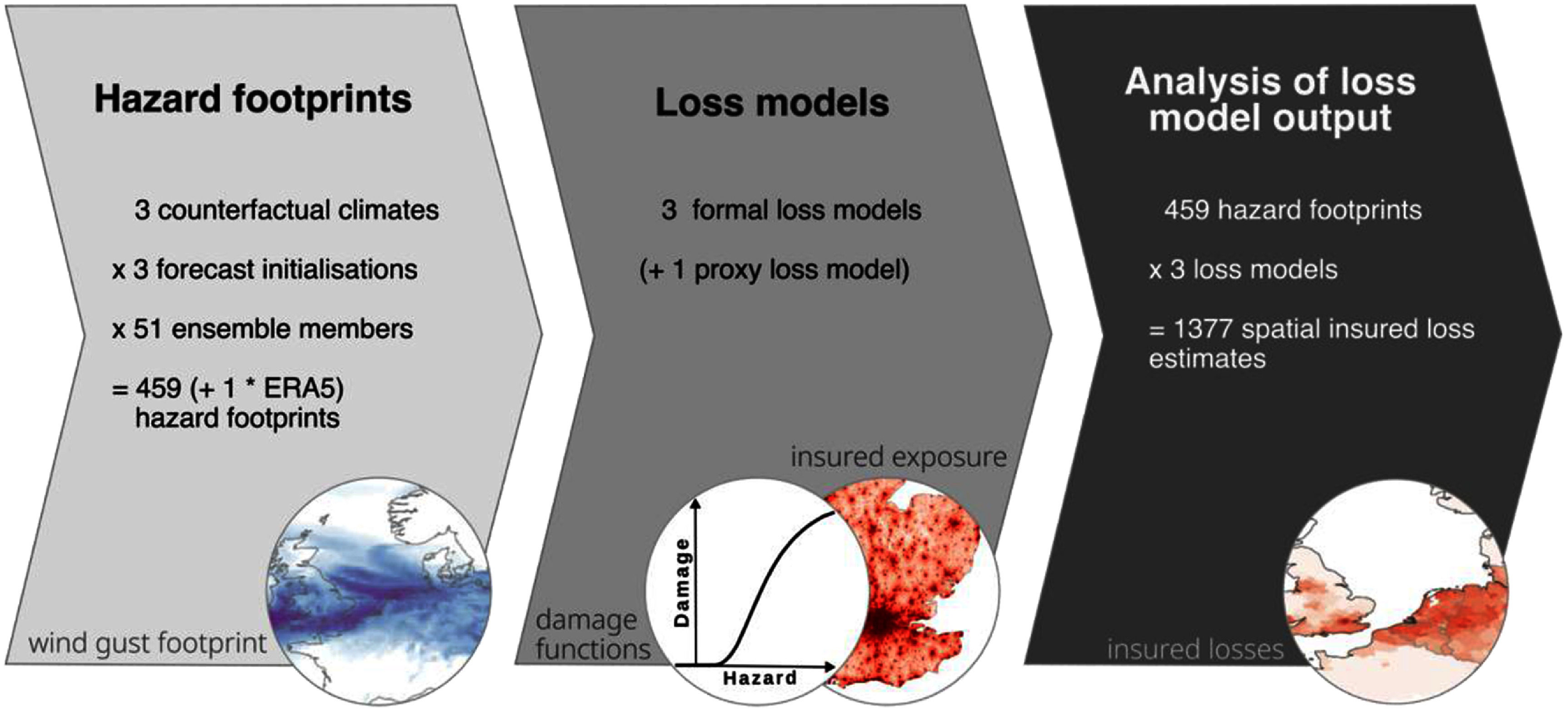
Modelling pipeline schematic. This shows the data flow, from hazard footprints, through the loss models (composed of damage functions and insured exposure data), into spatially explicit insured loss estimates. The damage function and exposure data shown are illustrative, with the exposure data taken from the LitPop dataset (Eberenz *et al* 2020). *CRESTA maps by GfK Geomarketing GmbH.*

**Figure 2.**
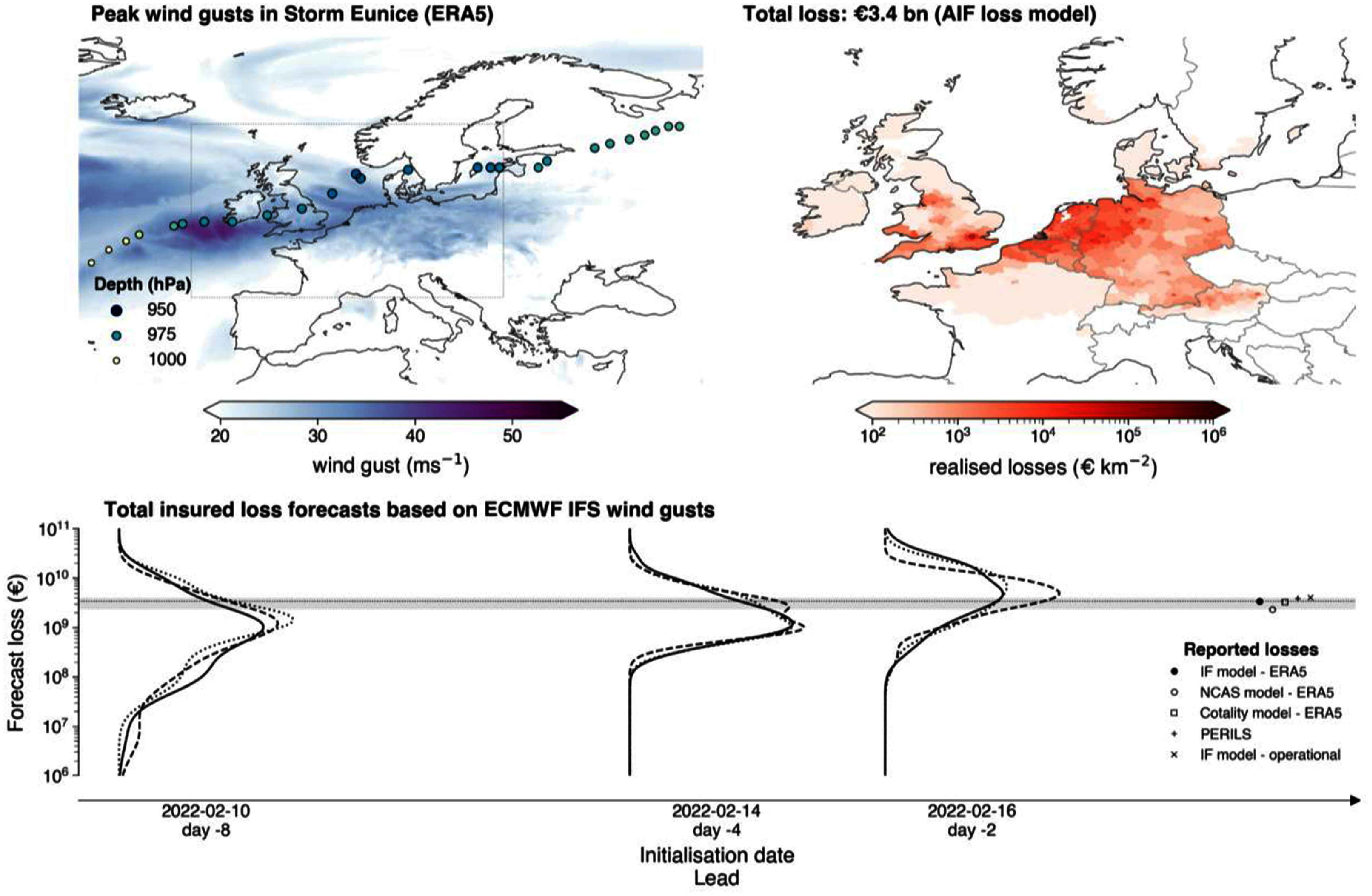
Storm Eunice, and its forecasted and estimated losses. Top left: storm Eunice represented by the ERA5 reanalysis. Shading indicates peak wind gusts during the storm. The coloured dots indicate the storm track and central pressure over the course of its transit across Europe. Top right: estimated losses from the AIF model forced by ERA5. Shading indicates the estimated loss density at a CRESTA zone scale. The title states the total estimated loss across the geographical domain covered by the AIF model results, which corresponds to the countries for which PERILS market exposure is available. Bottom: predicted insured loss PDFs across forecast initialisation dates from the combined IFS—loss model chain. The solid, dashed and dotted black lines indicate associated Gaussian kernel density estimates (KDE) from the AIF, NCAS, and Cotality models respectively. The horizontal shaded band indicates the range of insured loss estimates from five sources. The horizontal shaded grey line indicates the loss estimate from forcing the AIF model with ERA5 data. The markers on the right of the plot indicate individual insured loss estimates. *CRESTA maps by GfK Geomarketing GmbH.*

The hazard refers to the direct physical driver of a risk: such as the wind loading during a storm, or the inundation depth during a flood. Here, the hazard is provided through 72 hour maximum gust footprints extracted from the IFS ensemble forecast integrations (Dorland *et al* 1999). We use the 72 h definition for consistency with convention stemming from the ‘72-hour clause’: that damages incurred during a continuous 72 hour period are considered as a single event. The exposure refers to the value of the elements which are at risk. In this case, this is the spatially explicit value of insured buildings across Europe, and is modelled differently in the three models. An example of global exposure—a normalised version of which is used in the NCAS model—is shown in figure 2 in (Eberenz *et al* 2020), and as an inset in figure 1. The final component, vulnerability, refers to the propensity of those elements to sustain damage when affected by the hazard. Here, vulnerability is provided through damage functions (Prahl *et al* 2015), which typically relate the physical driver to the fraction of exposure lost. Again, these differ between the three loss models in terms of their specificity: the Aon IF and Cotality models distinguish between different classes of building (e.g. residential vs commercial), and are calibrated against empirical data including accounting for uncertainties; whilst the NCAS model uses a single cubic function. We would therefore expect losses to have the potential to be more non-linear with gusts in the Aon IF and Cotality models compared to the NCAS model. While the damage functions used in the Aon IF and Cotality models are proprietary and cannot be publicly shared, figure 2 in (Feuerstein *et al* 2011) provides additional context to this description by illustrating the estimated relationships between wind speeds and loss ratios for a variety of building construction types.

The output of each of these models is a spatial map, on the same grid as the exposure, of the modelled insured losses. We note that while some loss models link hazards to losses through statistical relationships typically applied at highly aggregated scales (i.e. ‘top–down’ or empirical; Murnane & Elsner 2012), the formal loss models used here all consider spatially explicit hazard footprints and exposure (Aznar-Siguan & Bresch 2019). For further information on the three models, see section S2 in the supplementary materials, and for a further introduction to this approach to loss modelling in general, please see (Mitchell-Wallace *et al* 2017).

The fourth ‘loss’ model, primarily used here for comparison, is the widely applied ‘loss index’ (Pinto *et al* 2012, Moemken *et al* 2024). This has been applied as a proxy for total storm loss in the absence of a formal loss model since it only requires gridded maximum wind speed (or gust) and population data for calculation (${v_x}$ and ${P_x}$ respectively; ${v_{98}}_x$ is the 98th climatological percentile). The population data used here is the gridded population of the World, Version 4 (University 2025). We use the original loss index form proposed by Pinto *et al* (2012):

\begin{equation*}LI = {\sum _x}{\left( {\dfrac{{{v_x}}}{{{v_{98x}}}}} \right)^3} \cdot {P_x} \cdot I\left( {{v_x},{v_{98x}}} \right) \cdot {L_x}, \end{equation*}

With

\begin{equation*}I\left({v_x},{v_{98x}}\right) = \left\{ \begin{array}{@{}l@{\quad}l} 0 &amp; {\mathrm{for}}\ {v_x} &lt; {v_{98x}} \hfill \\ 1 &amp; {\mathrm{for}}\ {v_x} &gt; {v_{98}}_x \hfill \\ \end{array} \right.\end{equation*}

and

\begin{equation*} {L_x} = \left\{\begin{array}{@{}l@{\quad}l} 0 &amp; \text{over sea} \\ 1 &amp; \text{over land} \end{array}\right.. \end{equation*}

We maintain a static loss model specification across the counterfactual climate states. This means that we do not consider changes to socioeconomic factors within our analysis. These could include effects such as reduced vulnerability between pre-industrial and present-day infrastructure, further infrastructure adaptation in the future, and changes to exposure over time. Our counterfactual scenarios are thus ‘climate counterfactuals’, isolating the impact of physical climate change on insured loss.

### 2.3. Statistical methods & uncertainty quantification

Each forecast ensemble produces an estimate of the probability distribution of loss outcomes (the ‘forecast uncertainty’). We then use this loss probability distribution to estimate specific metrics of interest that characterise the event, and compare these metrics across the different climates. The ‘event’ is characterised here in two ways. For the case of estimating changes in probability, we use the ERA5-estimated value for a given loss model. Where we estimate changes in the loss itself, we use the ERA5-estimated loss quantile within the present forecast ensemble. These different characterisations of the event allow us to estimate both the change in monetary loss, and the (conditional) change in the probability of experiencing a loss of the same magnitude.

Practically, we estimate the quantitative attribution results by parameterising the loss-probability relationship of a given forecast ensemble using a Weibull distribution. The distribution parameters are fit using L-Moments (Hosking 1990), and then used to estimate either the quantile of a given loss, or the loss of a given quantile. Since the ERA5-estimated value lies within the data range of all of the ensembles, no extrapolation beyond the data is required and the results are robust to the choice of distribution (see supplementary materials). Uncertainties in our estimates are quantified through a nonparametric bootstrap over ensemble members. This bootstrap is fully correlated across the different ensembles (i.e. the same set of ensemble members is used across all three counterfactual forecast ensembles for each bootstrap) to preserve the correlation structure imposed by the use of the same singular vector perturbations across the different counterfactual forecast ensembles.

## 3. Results

### 3.1. Forecast losses—model validation

A key step in weather attribution is to demonstrate that the model used is able to capture the necessary (physical or socioeconomic) processes (Palmer and Weisheimer 2018, Leach *et al* 2021). Here, as we are concerned with the impacts of storm Eunice, we demonstrate that the model used is suitable by verifying the loss forecasts it would have made. Previous work has already demonstrated the skill of IFS at representing the relevant physical processes through successful wind gust forecasts (Hewson *et al* 2022, Ermis *et al* 2024). We find that the total loss forecasts from both the coupled IFS-AIF and IFS-NCAS models are skilful, with similar conclusions for both, illustrated in figure 2. While the ensemble spread is significant, spanning several orders of magnitude, the ensemble captures the realised loss estimate, even at a lead time of over a week.

The loss models themselves are also able to accurately represent both the magnitude and patterns of loss. When forced by ERA5 reanalysis, the three loss models have total insured losses that lie between €2.3 bn and €3.4 bn, compared to an official estimate of €3.9 bn from PERILS (PERILS AG 2023). The Spearman correlation coefficients, calculated over inter-country loss variance against PERILS for the two models where country-specific losses were available (AIF and NCAS), are 0.95 and 0.87 respectively. From these three lines of evidence: forecast model validation previously done, loss model validation against observations, and successful combined model forecasts; we conclude that the modelling approach we have developed is suitable for the purpose of impact attribution.

### 3.2. Anthropogenic impact on realised losses

Within our experiment design, which considers ‘climate’ counterfactuals alone, any attributable anthropogenic impacts on the realised losses are a result of physical changes to the storm in the counterfactual climates. These physical changes were explored in Ermis *et al* (2024), and provide insight into drivers behind any attributable losses. They found that the peak wind gusts of the storm increased from the pre-industrial to the present to the future climate. In addition, across the counterfactual forecast ensembles, more explosive cyclones (those which reach a normalised deepening rate over 1) were found in the warmer climates. The increased wind speeds are associated with a higher vorticity at the centre of the storm in the warmer climate. Ermis *et al* demonstrated through a composite analysis that this arose from increased specific humidity and therefore latent heating in the warm conveyor belt resulting in greater vorticity production.

**Figure 3.**
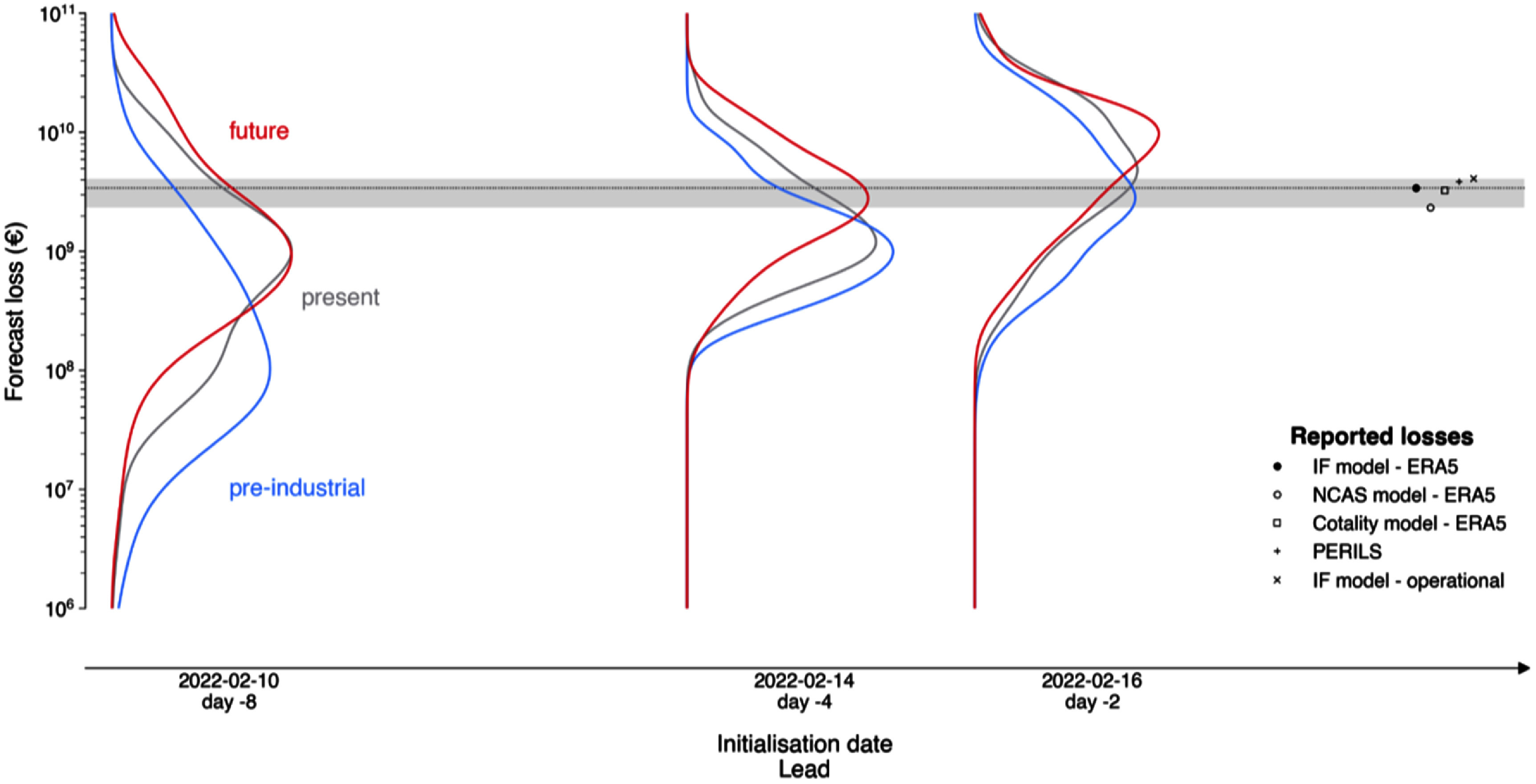
Counterfactual loss forecasts. The solid grey lines show present-day loss forecast PDFs (these are identical to the solid black lines in the lower panel of figure 2). The blue and red lines show counterfactual pre-industrial and future loss forecasts respectively. All other elements are identical to figure 2. For visual clarity, all forecasts shown are from the AIF model; the NCAS and Cotality models would result in a similar picture.

In accordance with the previous meteorological attribution, we find a clear anthropogenic signal within the counterfactual loss forecasts. This is illustrated in figure 3, which shows a clear upward shift in losses from the pre-industrial to the present, and present to the future forecast ensembles.

**Figure 4.**
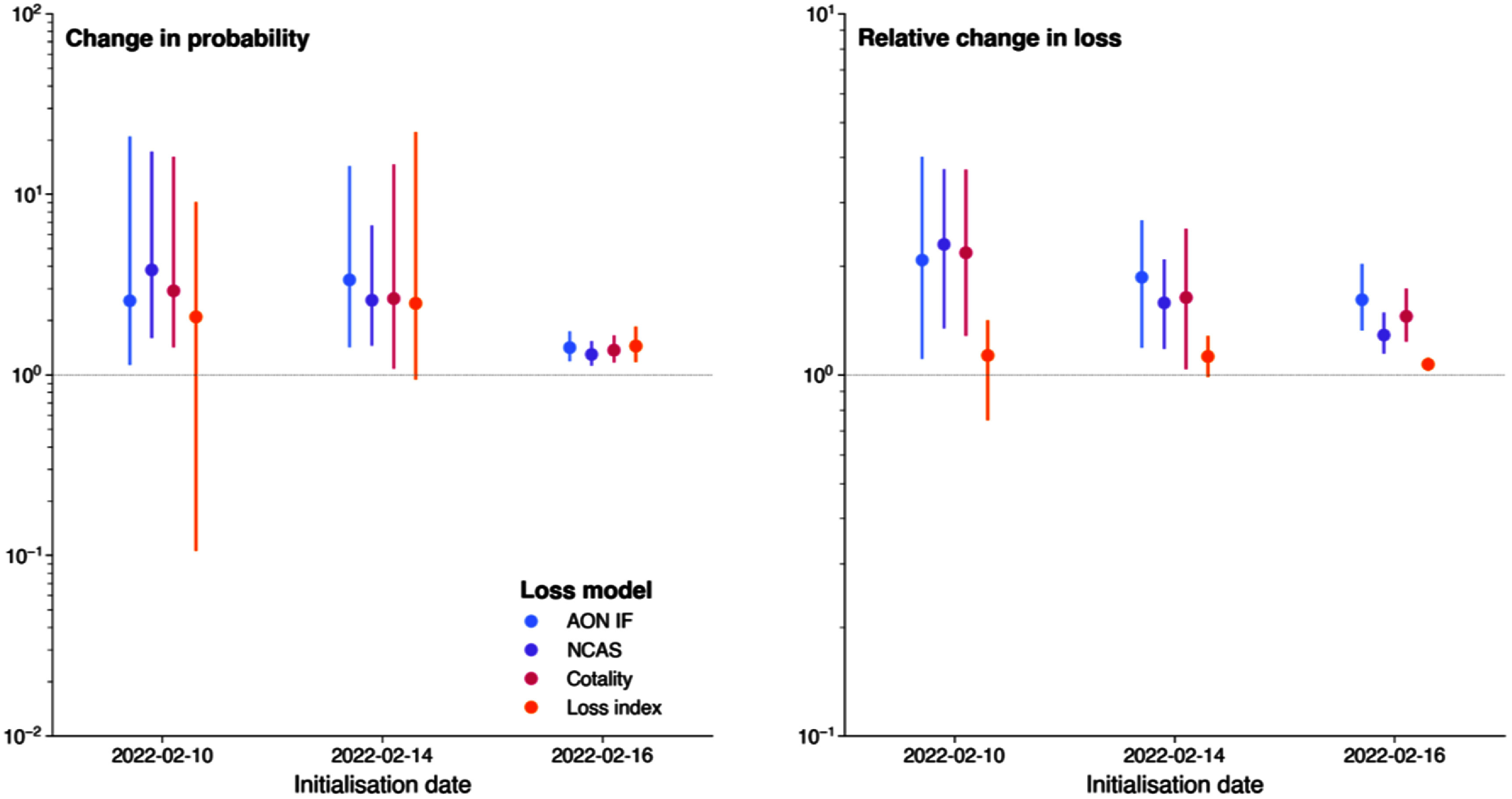
Anthropogenic influence on exceedance probability and relative changes in loss. Left: estimated change in probability of a Eunice-magnitude loss between pre-industrial and present-day forecasts. Right: estimated change in magnitude of loss, expressed as a multiplicative factor, between pre-industrial and present-day forecasts. In both panels, the dots indicate the central estimate, and lines indicate the 90% credible interval.

We present the attributable loss results in two flavours: as estimated changes in probability of a Eunice-magnitude loss event across the forecast ensembles; and as estimated change in magnitude of a factual forecast Eunice-probability loss event. We find that, conditional on the predictable component of the weather at the point of initialisation, the probability of a Eunice-loss storm is increased by a factor of 2.8 [1.2, 15]^9^9numbers in square brackets indicate a 90% credible interval estimated through a nonparametric bootstrap from the 51 forecast ensemble members which were used to drive each loss model. between a pre-industrial and present-day climate (figure 4). This result is consistent across the loss models and loss index, and across the two longer lead times. We find a smaller, but still significant change in probability for the shortest lead time, arising from the reduced adjustment period to the new climate states between model initialisation and storm onset. We find corresponding changes in loss of a factor of 1.9 [1.2, 3.1], equivalent to an absolute attributable loss of €1.8 bn. However, while this result is consistent across the three loss models, we obtain a significantly lower result of 1.1 [0.9, 1.3] when using the proxy loss index. This reduction in attributable change in losses arises from the considerably lighter tails of the forecasted proxy loss index distribution. This has been previously noted (Moemken *et al* 2024). While it is a good approximation of *ranked* losses, it significantly underestimates the quantitative change in loss. We would expect this result to hold generally for extreme windstorm losses; for less extreme windstorm losses the underestimation would likely be reduced.

We emphasise that our attribution results are conditional on the initial atmospheric state of the forecast model. This means that they are i) applicable to Eunice-like cyclones, and ii) that at shorter lead times may misrepresent the ‘full’ response as the forecast model atmosphere adjusts to the new climate state. This first caveat means that similar analyses performed on other windstorms may not yield the same quantitative or even qualitative conclusions, as the attributable response will depend on the precise physical processes and synoptic setup of the storm in question. The second caveat likely explains the reduction in estimated attributable changes in loss from the longest to shortest lead date, corresponding to the most to least adjusted forecasts.

### 3.3. How bad could it have been

In addition to the attribution results, which quantify the conditional change in probability or magnitude of an equal-size loss event to Eunice itself, our simulations can also be used to consider ‘downwards counterfactuals’ (Rye and Boyd 2022, Dixon *et al* 2025). This has previously been done in the context of the present-day climate, but our counterfactual forecasts allow us to further consider such downwards counterfactuals within past, present and future climates (Hazeleger *et al* 2015). In this section, we focus on the results from the Aon IF loss model, noting the extreme tail of the NCAS model is lighter, with the Cotality model between the other two. The extremes within the NCAS model are significantly lower, though still represent exceptional loss events. This may arise due to the increased potential for non-linearity in the Aon IF model from its more specific and calibrated damage functions. There is therefore considerable epistemic uncertainty in these ‘unprecedented’ loss events and including additional loss models, whether open-source or proprietary, represents an important aspect of future work in this direction. Hence we primarily present losses relative to the estimated Eunice loss within the corresponding model to attempt to account for some, but certainly not all, of these epistemic uncertainties.

**Figure 5.**
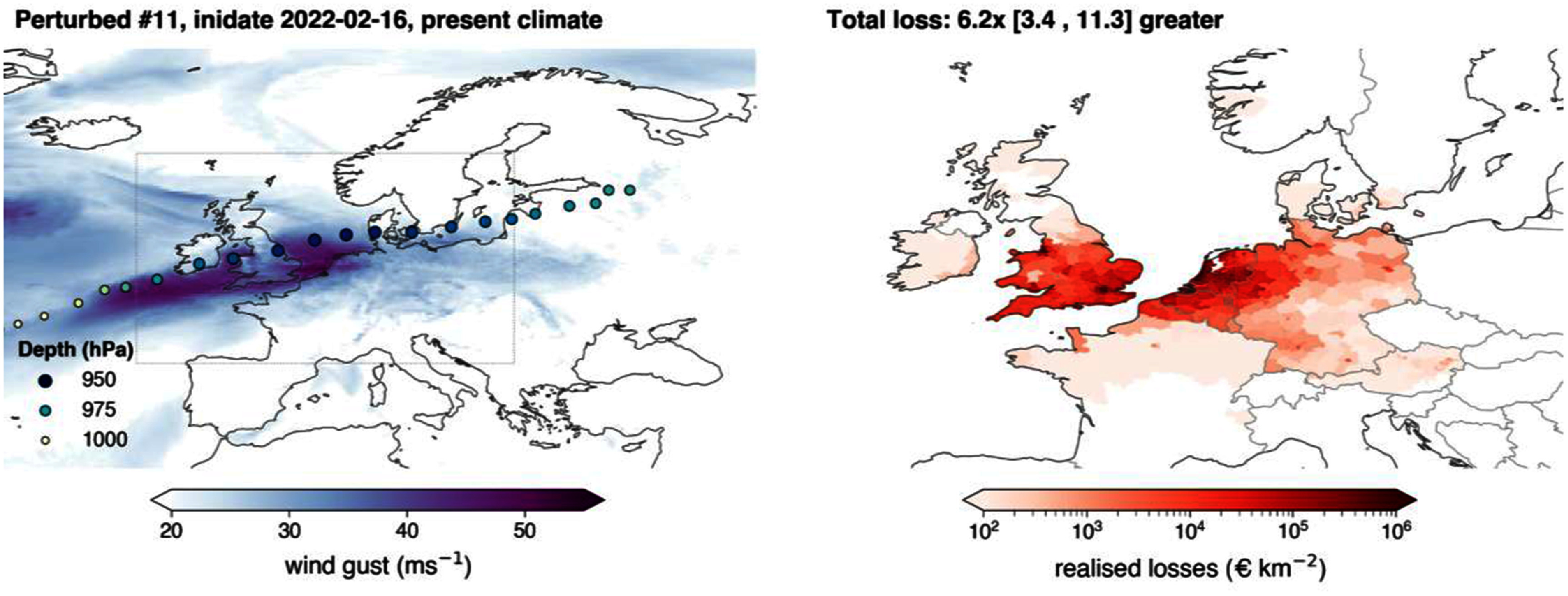
A plausible, but unrealised present-day Eunice storyline and its estimated losses. As the top panel of figure 2, but for ensemble member #11 of the present-day forecast initialised on 2022-02-16. The title in the right panel states the central total insured loss across the three loss models as a multiple of the loss estimated from the ERA5 footprint, with numbers in brackets representing the minimum and maximum losses across the models. *CRESTA maps by GfK Geomarketing GmbH.*

We find two scenarios of note within our ensembles. Within the present climate forecast ensemble, one member in the AIF model ensemble reaches an estimated insured loss of over 10x greater than the loss based on an ERA5 footprint (figure 5). This would represent an unprecedented loss for the insurance industry in the context of the European windstorm peril; the highest loss event historically was Daria, reaching an insured loss of €12.7 bn (J. F. Roberts *et al* 2014; inflated to 2022 values; equal to 3.3x the PERILS Eunice estimate). We note that some of this increase will be due to changes to exposure throughout Europe—if Daria were to reoccur today, the losses may well be over and above that expected from inflation alone. Some of the increased loss due to exposure change would, however, be offset by changes to the vulnerability through improved building codes and construction practices. We also note that this magnitude loss represents the very top-end of loss events represented within the event set of the operational Aon IF European windstorm natural catastrophe model. Based on a previous comparison of other models, a loss event of this magnitude is at least a 1-in-250 year loss event, and exceeds the estimated 400 year loss event in the majority of the participating vendor models (Waisman 2015). Although the other loss models are more conservative with regards to the extreme tail of loss, both contain ensemble members with exceptional loss events well above the value realised by Eunice.

**Figure 6.**
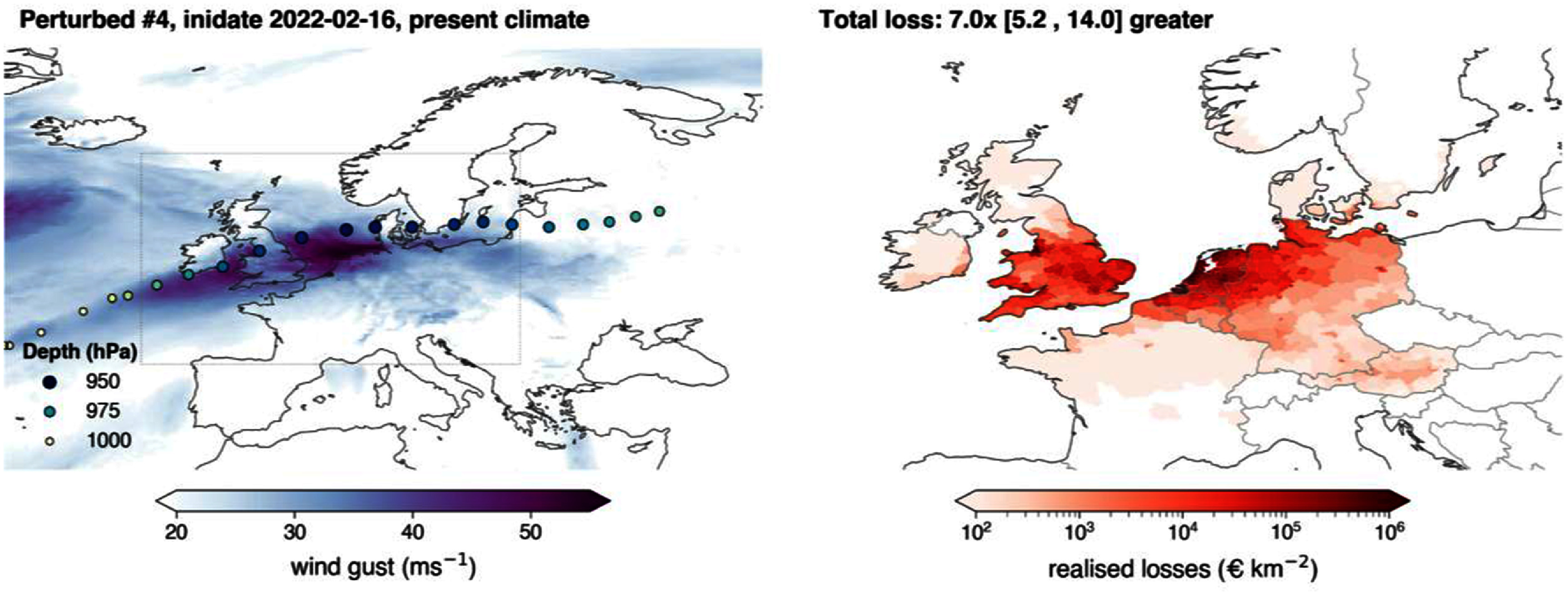
A plausible future Eunice storyline and its estimated losses. As the top panel of figure 2, but for ensemble member #4 of the counterfactual future climate forecast initialised on 2022-02-16. *CRESTA maps by GfK Geomarketing GmbH.*

We also find that the climate responses detected in wind intensity (Ermis *et al* 2024) and loss are also reflected in such downwards counterfactual events. Within the ‘future’ climate forecast, which represents an equal and opposite change to the pre-industrial climate, the top loss event in the AIF ensemble reaches 14x the estimated actual Eunice loss; in the NCAS and Cotality models it is 5.2 and 7.0 times greater respectively (figure 6). In this ‘future super-Eunice’ windstorm, the cyclone deepens continually during its transit of Europe until reaching Denmark, achieving a peak depth of 955 hPa, compared to the peak depth of 975 hPa attained by the realised Eunice. The wind gusts are correspondingly higher, in excess of 55 ms^−1^ over parts of the North Sea. The Netherlands bears much of the impacts in terms of insured loss estimated for this AIF model scenario: €30.3 bn of the total, equivalent to over 3% of its annual GDP (‘GDP (current US$)’ n.d.). Although these exposures are not considered in this study, we note that both of these counterfactual windstorms reach peak intensity over an area where a considerable amount of offshore wind infrastructure is located. Potential applications of these findings are discussed below. Again, we note that the other loss models are more conservative with respect to the magnitude of these extreme losses, but the pattern is consistent across all models: the top loss event in the future counterfactual forecast is greater than the top event in the present forecast.

## 4. Discussion

Here, we have presented a conditional impact attribution of storm Eunice, the first of its kind for European windstorm losses (Mudhar *et al* 2024). This attribution is based on coupling a state-of-the-art operational medium-range weather forecast model, which is demonstrably capable of capturing the key physical processes (Ermis *et al* 2024), to three loss models: two proprietary models used operationally within the (re)insurance sector, and an open-source model developed in academia (Kumar *et al* 2023). We find a detectable anthropogenic influence on the losses incurred during the storm likely greater than €1.6 bn compared to what they would have been in a pre-industrial climate.

The attribution presented is conditional. Since the forecast model is initialised within the window of predictability, the statements made are conditioned on the predictable component of the weather at the time of initialisation. This means that the attribution presented is primarily to the thermodynamic response to climate change (Shepherd 2016), and will not include the impacts of climate change on longer-timescale modes of variability, such as the NAO. We note that this forecast-based approach does allow for more flexibility in terms of the dynamical response than other such conditional ‘storyline’ approaches since we impose no constraints on the dynamical state as is often done in these other approaches: we allow the model to evolve freely. However, this potential dynamical response is limited to what can be materialised during the time between forecast initialisation and the event itself, which in this study is at most 8 d. We suggest that counterfactual seasonal forecasts could be used in an analogous approach to understand climate change impacts on important longer timescale modes of variability.

### 4.1. The importance of impact attribution

A key advantage of the forecast-based approach used here is that the underlying forecast model is already used operationally across a wide variety of impact modelling pipelines, and has extremely low bias compared to conventional approaches using climate models. As such, we were able to force the loss models using direct outputs from the weather forecast model without any bias-correction, statistical or otherwise, that might introduce additional structural uncertainties to the results.

We have also demonstrated the importance of using impact models over meteorological proxies. Although such proxies can provide some guidance as to the relative impact of different events, we found that a widely used proxy for loss (Pinto *et al* 2012) fails to capture the relative changes in the tail of the forecast loss distribution, which results in a considerably underestimated attributable response compared to the three loss models. This arises due to the lighter tails of the proxy distribution. We argue that this result will likely hold more generally across other socioeconomic impacts and therefore caution strongly against drawing conclusions about impacts from purely meteorological indices. True impact attribution using well-calibrated impact models is critical to ensure robust results and avoid both under- or overestimating any climate change responses as a result of non-linearities in translation between hazard and impact.

### 4.2. Academic—industry partnerships

An important insight from this study is on the value of academic-industry collaboration. These could, and should, play a vital role in understanding the impact of climate change on extreme weather risk. While academia typically has a primary focus on the physical aspects of climate change (using the conventional definition of risk, this would be the ‘hazard’ component), industry—in this case the natural catastrophe (re)insurance sector—can provide resources due to their focus, and more developed understanding of the other aspects: exposure and vulnerability in the context of economic damages.

This study has demonstrated the considerable practical advantages of such collaboration. It would be both time-consuming and challenging to attempt to replicate the work done in the other sector, with each providing added value to the other. We therefore strongly encourage future collaborations; though acknowledge the potential challenges of meeting increasing open-source requirements for peer-reviewed publication with proprietary impact models.

### 4.3. Future directions

There are a number of potential directions that could be taken forward from this work. One would be to expand the scope beyond a single storm, and carry out conditional impact attribution analyses for several storms, or for a season of storms, similar to meteorological attribution work previously done for tropical cyclones (Reed *et al* 2022). This would provide a more general picture of human influence on European windstorms as the results here are specific to the physical processes and synoptic conditions during storm Eunice and do not generalise to all windstorms. Another similar direction would be to reduce the level of conditionality in the counterfactual forecasts by using a seasonal forecasting ensemble (Leach *et al* 2024) or even a decadal prediction system. This would enable the dynamical response to climate change, including changes to windstorm frequency, to be quantified better; and could be synthesized with the counterfactual medium-range forecasts used here to provide a ‘seamless’ attribution assessment. An alternative direction would be to understand how projected changes in the exposure and vulnerability components of the risk would affect our results.

There also remain limitations in the experiment design to be addressed in future work. As previously mentioned, a key caveat in the results presented is that the forecast model atmosphere adjusts freely to the counterfactual initial states used in the pre-industrial and future simulations. This avoids issues present in alternative approaches to generate counterfactual atmospheric states including: inconsistency between the prescribed perturbation and specific atmospheric state at the time of the event of interest (Brogli *et al* 2023); or the introduction of a potential ‘drift’ component in the estimated response that is difficult to separate from the true response in nudged model simulations (Benítez *et al* 2022). A methodology to estimate state-specific atmospheric perturbations would be a major step forwards; one promising direction towards this is such ‘counterfactual’ reanalyses (Hawkins *et al* 2023). Another important limitation is the anthropogenic forcings considered. The counterfactual forecasts used here consider only the response to atmospheric CO_2_ in the boundary conditions. However, other anthropogenic forcings, such as aerosols, may have had an influence on European windstorm risk over the historical period. Limited research exists on the explicit response of windstorm risk to aerosols, but aerosol influences on the synoptic drivers of the storm track have been shown (Booth *et al* 2012, Pausata *et al* 2015, Dagan *et al* 2020). Explicit quantification of this response represents an important gap: progress towards this could be made by future counterfactual forecast simulations that include perturbations to aerosol concentrations in addition to greenhouse gas.

A separate limitation of the experiment design to address in future work would be to implement this forecast-based approach to attribution within multiple forecast models, so as to estimate the structural model uncertainty of the response to climate change. While the conditional nature of the simulations performed here and the ability of IFS to capture the physical mechanisms of the storm provides confidence in our single-model analysis (at least when compared with equivalent single-model studies that use conventional probabilistic approaches to attribution), other models may respond differently to climate forcing, and a multi-model assessment is an important direction for future development of this approach.

Our final suggested future direction relates to potential application of the loss forecast ensembles here. Within our ensembles, there exist several exceptionally high impact loss events. These could be used to inform stress testing across multiple sectors, including finance [both (re)insurance and banking], emergency planning, and infrastructure design. Current tests are often based on statistically inferred high impact events: ‘risk maps’ (European Central Bank 2022, Monetary Authority of Singapore 2022), and are not necessarily physically consistent. We argue that introducing such fully physically plausible events into existing stress testing frameworks would represent a significant step forward. It would also allow for the consideration of physically consistent compounding hazards, or downward counterfactual analysis such as is done in the insurance sector due to the intrinsic link to a real-world event (Rye and Boyd 2022). Alternatively, these could be used for the validation of extreme tail events produced by the hazard component of natural catastrophe models, especially relevant for those dependent on primarily statistical modelling frameworks.

### 4.4. Concluding remark

We have found that if a Eunice-like storm were to occur in a warmer climate, it would not only be stronger, but would cause considerably higher insured losses. Due to the highly non-linear relationships between hazard magnitude and local damage, even relatively small forced changes in the hazard can result in large changes to the total loss. Being able to accurately quantify how financial losses driven by extreme weather will be affected by climate change is of critical importance to a wide variety of stakeholders for adaptation planning and design. We suggest that this work, which combines counterfactual forecasts from a state-of-the-art weather model with operational loss models used in the insurance sector, sets out a promising framework that could be used across a wide range of hazards.

## Data Availability

Counterfactual and factual forecast data are available from the mars archive at ECMWF under anonymous access: Insured loss forecast data output by the loss models is available for the NCAS and AIF models only (Leach *et al* 2025). Cotality model output is not available due to its proprietary nature. Supplementary information available at: https://doi.org/10.1088/1748-9326/ae886a/data1.
